# Identification of genomic region(s) responsible for high iron and zinc content in rice

**DOI:** 10.1038/s41598-019-43888-y

**Published:** 2019-05-31

**Authors:** Shilpi Dixit, Uma Maheshwar Singh, Ragavendran Abbai, T. Ram, Vikas Kumar Singh, Amitava Paul, P. S. Virk, Arvind Kumar

**Affiliations:** 1 International Rice Research Institute (IRRI), South-Asia Hub, ICRISAT Campus, Hyderabad, 502324 India; 2 Indian Institute of Rice Research (IIRR), Hyderabad, 500030 India; 3 Visva-Bharati University, Santiniketan, West Bengal 731235 India; 4 HarvestPlus, ICRISAT Campus, Hyderabad, 502324 India; 5 International Rice Research Institute, DAPO BOX 7777, Metro Manila, Philippines

**Keywords:** Plant breeding, Plant breeding

## Abstract

Micronutrient especially iron and zinc-enriched rice hold immense promise for sustainable and cost-effective solutions to overcome malnutrition. In this context, BC_2_F_5_ population derived from cross between RP-Bio226 and Sampada was used to localize genomic region(s)/QTL(s) for grain Fe (iron) and Zn (zinc) content together with yield and yield-related traits. Genotyping of mapping population with 108 SSR markers resulted in a genetic map of 2317.5 cM with an average marker distance of 21.5 cM. Mean grain mineral content in the mapping population across the two seasons ranged from 10.5–17.5 ppm for Fe and 11.3–22.1 ppm for Zn. Based on the multi-season phenotypic data together with genotypic data, a total of two major QTLs for Fe (PVE upto 17.1%) and three for Zn (PVE upto 34.2%) were identified. Comparative analysis across the two seasons has revealed four consistent QTLs for Fe (*qFe*_*1.1*_, *qFe*_*1.2*_, *qFe*_*6.1*_ and *qFe*_*6.2*_) and two QTL for Zn content (*qZn*_*1.1*_ and *qZn*_*6.2*_). Additionally, based on the previous and current studies three meta-QTLs for grain Fe and two for grain Zn have been identified. In-silico analysis of the identified QTL regions revealed the presence of potential candidate gene(s) such as, *OsPOT*, *OsZIP4*, *OsFDR3*, *OsIAA5* etc., that were previously reported to influence grain Fe and Zn content. The identified QTLs could be utilized in developing high yielding, Fe and Zn denser varieties by marker assisted selection (MAS).

## Introduction

Rice (*Oryza sativa* L.) is one of the major food crops which is consumed globally. According to a recent report, rice is cultivated over 161 million hectares accounting for about 488.3 million tons milled rice production world wide^1^. Improvement in nutritional and agronomic traits of rice is bound to affect a sizeable population since it is a primary source of livelihood. Increasing the nutritive value of rice is considered as one of the promising strategies to overcome malnutrition, as its per-capita consumption ranges between 62–190 kg/year in rice consuming countries^2,3^.

Fe, Zn, iodine and vitamin-A are the micronutrients considered essential to improve human health and reduce the risk of malnutrition^4^. The demand for most of these nutrients can be met through consumption of cereals, particularly rice due to its staple role in food consumption provided the content of micronutrient in rice can be improved^4^. Deficiencies in bioavailable Fe, Zn and other essential cation minerals in human food, cause mineral malnutrition and affect a large proportion of the world population. Fe and Zn deficiencies are among the most prevalent micronutrient deficiencies in humans, affecting two billion people and causing more than 0.8 million deaths annually^5^.

To ameliorate micronutrient malnutrition associated health problems, researchers are working to enhance grain Fe and Zn content of staple cereal crops by different breeding approaches. Biofortification of crops through breeding is a cost effective and sustainable strategy to solve micronutrient malnutrition for people living in developing countries that cannot afford Fe and Zn fortified foods, by supplementation of Fe and Zn into their staple diets. Several QTLs associated with high grain Fe (19 QTLs) and Zn (13 QTLs) content have been mapped in rice^6–9^. Meta-QTL study for grain Fe and Zn traits of already reported QTLs can provide better understanding of the QTL effect in different genetic backgrounds and to locate major effect QTLs. This analysis can be considered as part for validation of QTLs by knowing the confidence interval (CI) for the already reported QTLs^10^. Combining different studies relating to QTLs for grain Fe and Zn content to identify meta-QTL is a very promising approach to understand the QTL effect in different genetic backgrounds and to locate QTL position on the consensus map. The genomic regions identified through meta-QTL analysis can be directly utilized in crop improvement.

QTL mapping provides opportunities for identification of the genomic region(s) associated with the targeted traits by combining genome information with phenotyping^11^. Subsequently identified genomic region(s)/QTLs/genes could be deployed in the breeding programs through marker-assisted selection (MAS). Therefore, in the present study, SSR based genetic map of a backcross-derived mapping population crossed between RP-Bio226 (Fe 11.3 ppm and Zn 21.1 ppm) × Sampada (Fe 10.5 ppm and Zn 18.3 ppm) were generated. Mapping population together with parental lines was phenotyped for grain Fe and Zn content together with yield and yield-associated traits for two seasons. Subsequently, detailed analysis of genotypic data together with phenotyping data have provided consistent genomic region(s)/genes associated with high grain Fe and Zn content in rice along with yield and yield associated traits.

## Results

### Mapping populations and phenotypic evaluation

Mapping population (RP-Bio226 × Sampada) was developed at Indian Institute of Rice Research (IIRR), Hyderabad using backcross breeding method. A total of 111 BC_2_F_5_ individuals along with their parental lines and check (MTU 1010) were phenotyped for two seasons (DS2015 and WS2015) at the International Rice Research Institute-South Asia Hub (IRRI-SAH), ICRISAT, Patancheru (78° 16′ longitude, 17° 32′ latitude and 540 m above sea level) using alpha lattice design.

**Figure 1:**
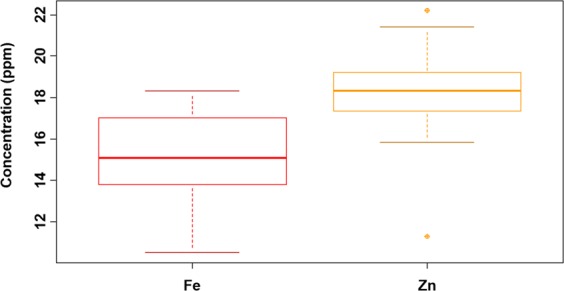
Box plot depicting the phenotypic variance for grain Fe and Zn content. Mean values for Fe and Zn content across DS2015 and WS2015 in BC_2_F_5_ population derived from a cross between RP-Bio226 and Sampada was utilized to develop boxplot. Significant variation among the population was observed for both the micro-nutrients.

Parents of the mapping population showed phenotypic differences for grain Fe and Zn content. The parent Sampada showed 10.5 ppm of grain Fe and 18.3 ppm of grain Zn for both seasons (mean data of DS2015 and WS2015), whereas another parent, RP-Bio226 has grain Fe 11.3 ppm and grain Zn content as 21.1 ppm. Considerable phenotypic variation was also found in mapping population for grain Fe and Zn during two crop seasons. Grain iron content in population ranged from 9.1–16.7 ppm in DS2015 and 11.7–18.3 ppm in WS2015, whereas grain zinc ranged from 12–23.1 ppm in DS2015 and 10.6–21 ppm in WS2015 **(**Fig. 1**)**. The range of phenotypic variations observed for the targeted trait indicates that most likely several genes contribute to iron, zinc homeostasis, and accumulation in grains. Further, it indicates that the developed genetic material is a suitable source for QTL mapping (Table 1). Also, the frequency distribution for yield and yield-related traits shows that most of the traits followed a normal distribution (Fig. S1).

**Table 1:** Descriptive statistics of seven agronomic traits in the parents and BC_2_F_5_ population derived from cross between RP-Bio226 × Sampada.

Season	Description	DFF	PH	PL	NT	GY	Fe	Zn
DS2015	RP-Bio226	133	92	22	13	4231	10.4	23.4
	Sampada	130	89	23	9	5108	9.5	19.9
	Population (range)	90–135	81–118	18–26	7–19	1121–8812	9.7–16.7	12–23.1
WS2015	RP-Bio226	128	92	21	10	4930	12.2	19.1
	Sampada	125	82	22	9	5663	11.2	16.8
	Population (range)	88–133	77–111	20–26	8–16	1383–8333	10.4–17.5	10.6–21

DFF = days to 50% flowering; PH = plant height (cm); PL = panicle length (cm); NT = number of tillers; GY = grain yield (kg/ha); Fe = iron content (ppm); Zn = zinc content (ppm)

### Correlation analysis of grain Fe and Zn with agronomical traits

A total of 28 pairwise combinations were formed for seven traits of which five coefficient of correlation estimates were found to be significant at 1% level and three combinations were significant at 5% level (Table 2). Pearson correlation results indicated there were significant correlations between phenotypic traits (DFF; PH; PL; NT; GY) along with grain iron and zinc in rice. It was found that phenotypic trait; grain yield and number of tillers were negatively correlated to grain iron and zinc whereas, plant height, panicle length was found to show significant positive correlations. Highly significant positive correlation was observed between grain iron and zinc trait.

**Table 2:** Correlation of the Fe and Zn content with yield and yield related traits in RP-Bio226 × Sampada population.

Trait	DFF	PH	PL	NT	GY	Fe	Zn
DFF	1						
PH	−0.07	1					
PL	−0.15	0.31**	1				
NT	0.19*	−0.32**	0.21*	1			
GY	−0.08	−0.13	−0.04	0.20*	1		
Fe	−0.03	0.04	0.06	−0.13	−0.09	1	
Zn	0.01	0.26**	0.05	−0.02	−0.41**	0.43**	1

*Significant at the 0.05 level (2-tailed).

**Significant at the 0.01 level (2-tailed).

DFF: days to 50% flowering; PH: plant height; PL: panicle length; NT: number of tillers; GY: Grain yield; Fe: grain iron content (ppm); Zn: grain zinc content (ppm).

### Microsatellites- based genetic map

Identification of enough markers revealing polymorphism among the parental lines is a prerequisite for the construction of a genetic linkage map. In this study, parental polymorphism survey was carried out between (RP-Bio226 and Sampada) using a total of 1000 SSR markers spanning across all the 12 rice chromosomes of rice and among that, 108 SSR markers were found to be polymorphic. The SSR marker sequences were taken from gramene rice genome browser (http://www.gramene.org/). The segregation distortion was analyzed for all the 108 SSR loci using *X*^2^ test and all showing normal Mendelian segregation distributed over all the 12 rice chromosomes were used for the construction of molecular linkage map using QTL IciMapping software^12^. Relatively, more number of polymorphic markers were found on Chr 08 with a polymorphism of 24.5% while lowest level of polymorphism was found in Chr 05 (4.41%). Chromosome-wise marker density varied from 2.4 cM/marker on Chr 03 to 32.4 cM/marker on Chr 07 (Table 3).

**Table 3:** Details of the linkage map constructed using RP-Bio226 × Sampada BC_2_F_5_ population.

Chr	Total markers	Number of polymorphic markers	Polymorphism (%)	Map length (cM)	Marker density (cM/marker)
01	104	13	12.5	342.5	26.3
02	74	14	18.9	381.7	27.2
03	88	14	15.9	215.2	2.4
04	73	6	8.21	73.6	12.3
05	68	3	4.41	17.2	5.7
06	64	14	21.8	309.8	22.1
07	35	6	17.1	194.7	32.4
08	56	13	24.5	220.7	16.9
09	74	9	12.2	169.3	18.8
10	47	3	6.38	47.4	15.8
11	70	8	11.4	210	26.3
12	34	5	14.7	135.4	27.1
Average	65.5	9	14	193.1	19.4

### QTL analysis

The phenotypic and genotypic data generated from BC_2_F_5_ populations were used for QTL analysis using composite interval mapping (CIM) in QTL cartographer V2.5^13^. The QTLs identified in both season were considered as consistent QTLs. Further, QTLs showing >10% PVE were considered as major QTLs, otherwise considered as minor QTLs.

### QTLs for grain mineral content

**Figure 2:**
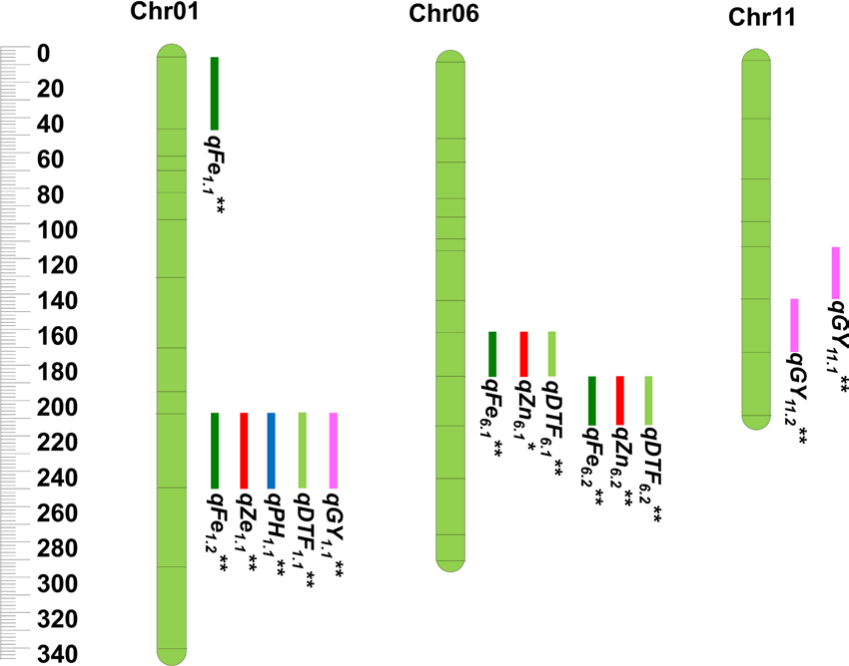
SSR based genetic map and distribution of QTLs associated with grain Fe and grain Zn (RP-Bio226 × Sampada). The scale on the left indicates genetic distance (centiMorgan; cM as unit). The black lines in the linkage groups represent the genetic position of the markers. A total of three linkage groups, namely Chr 01, Chr 06 and Chr 11 possess 14 QTLs for grain Fe, Zn and yield and yield-related traits. Note: *represent QTL identified in one season and ** represents stable QTL (identified in both seasons, 2015 DS & 2015 WS).

A total of two major QTLs were identified in this study for grain Fe on Chr 01 with PVE ranging from 14 (*qFe*_*1.2*_) to 17.1% (*qFe*_*1.1*_) in DS2015, while no major QTL was detected in WS2015 (Table 4, Fig. 2). Comparative analysis of the two season datasets revealed fours consistent QTLs (appeared in two season), namely, *qFe*_*1.1*_ (17.1% of PVE), *qFe*_*1.2*_ (14% of PVE), *qFe*_*6.1*_ (9.8% of PVE) and *qFe*_*6.2*_ (5.1% of PVE) attributing to grain Fe content.

**Table 4:** Mapping of QTLs governing grain Fe and Zn content, PH, DTF and GY using RP-Bio226 × Sampada BC_2_F_5_ population.

Trait name	QTLs	Chr.	Position (cM)	Marker interval	Right marker	Left marker	LOD DS2015	PVE (%) DS2015	Additive DS2015	LOD WS2015	PVE WS2015 (%)	Additive WS2015
Fe	*qFe*_*1.1*_	01	27	RM562-RM11943	RM562	RM11943	10	17.1	0.11	4.2	4.8	0.6
	*qFe*_*1.2*_	01	244	RM294A-RM12276	RM294A	RM12276	7.8	14	1.11	2.5	9.1	0.9
	*qFe*_*6.1*_	06	176	RM8226-RM400	RM8226	RM400	2.6	6.6	−0.04	3.7	9.8	−0.01
	*qFe*_*6.2*_	06	180	RM400-RM162	RM400	RM162	7.6	5.1	0.65	2.6	1.4	1.2
Zn	*qZn*_*1.1*_	01	244	RM294A-RM12276	RM294A	RM12276	2.6	14.3	3.2	3.7	9.8	0.8
	*qZn*_*6.1*_	06	176	RM8226-RM400	RM8226	RM400	3.9	34.2	1.21	—	—	—
	*qZn*_*6.2*_	06	180	RM400-RM162	RM400	RM162	3.5	2.9	0.06	2.8	14	1.12
PH	*qPH*_*1.1*_	01	224	RM294A-RM12276	RM294A	RM12276	2.5	21	−10.09	6.2	14.3	0.01
DTF	*qDTF*_*1.1*_	01	325	RM294A-RM12276	RM294A	RM12276	9.6	17.4	1.12	4.8	11.2	0.8
	*qDTF*_*6.1*_	06	215	RM8226-RM400	RM8226	RM400	11.8	22.6	0.97	5	14.7	−0.17
	*qDTF*_*6.2*_	06	180	RM400-RM162	RM400	RM162	10	10.8	3.4	3.8	6.8	1.18
GY	*qGY*_*1.1*_	01	305	RM294A-RM12276	RM294A	RM12276	3.6	11	1.12	5.3	29.8	0.89
	*qGY*_*11.1*_	11	135	RM552-RM21	RM552	RM21	3	12.2	−1.01	5.6	16.4	−0.13
	*qGY*_*11.2*_	11	152	RM21-RM224	RM21	RM224	3.4	11.2	0.96	5.7	4.8	2.1

Fe: grain iron content (ppm); Zn: grain zinc content (ppm); PH: plant height (cm); DFF: days to 50% flowering; GY: grain yield(kg/ha); cM: centiMorgan; PVE: Phenotypic variance explained.

Similarly, QTL analysis was performed to uncover the genetic basis of zinc in grains using composite interval mapping (CIM), which resulted in identification of two major QTLs (*qZn*_*1.1*_*, qZn*_*6*.1_) for grain zinc with PVE ranging from 14.3 (*qZn*_*1.1*_) to 34.2% (*qZn*_*6.1*_) with LOD value of 2.6 to 3.9 respectively for DS2015. However, in WS2015 only one major QTL (*qZn*_*6.2*_) with PVE 14% and LOD score of 2.8 was identified. Upon comparison of data across seasons, two stable QTLs namely, *qZn*_*1.1*_ (14.3% of PVE) and *qZn*_*6.2*_ (14% of PVE) were identified. After appropriate validation, the identified major and stable QTLs can be further utilized in the breeding program.

### QTLs for yield and yield-related traits

QTL analysis for plant height revealed a major QTL (*qPH*_*1.1*_) on Chr 01 with PVE 21% and LOD value 2.5 in DS2015 and PVE of 14.3% with LOD 6.2 in WS2015. Three major stable QTLs for DFF were mapped, one QTL (*qDTF*_*1.1*_) on Chr 01 with 17.4% of PVE and LOD value 9.6 in DS2015 and 11.2% PVE with LOD score of 4.8 in WS2015; two QTLs namely, *qDTF*_*6.1*_ and *qDTF*_*6.2*_ on Chr 06 with PVE ranging from 10.8–22.6% and LOD value ranging from 10–11.8 were observed in DS2015 and PVE of 6.8–14.7% with LOD value 3.8–5 in WS2015. For grain yield, three major QTLs (*qGY*_*1.1*,_ *qGY*_*11.1*,_ *qGY*_*11.2*_) were identified on Chr 01 and Chr 11 with PVE 11, 12.2, 11.2% and LOD value 3.6, 3.0 and 3.4 respectively in DS2015, whereas two major QTL (*qGY*_*1.1*,_ *qGY*_*11.1*_) on Chr 01 with PVE 29.8% (LOD value of 5.3) and Chr 11 with PVE 16.4% (LOD value of 5.6) was identified in WS2015. Comparative analysis of datasets for grain yield QTLs of both seasons, revealed three major and stable QTLs.

### Co-localized QTL for grain iron and zinc content with yield-related traits

Interestingly, it was found that grain Fe, Zn, PH, DFF and GY QTLs were co-localized. Genomic region flanking RM294A and RM12276 marker on Chr 01 localized five important QTLs namely *qFe*_*1.2*_ (PVE % of 14), *qZn*_*1.1*_ (PVE % of 14.3), *qDTF*_*1.1*_ (PVE of 17.4%), *qPH*_*1.1*_ (PVE % of 21) and *qGY*_*1.1*_ (PVE of 11%). Also, on the same chromosome three genomic regions were shared for grain Fe (*qFe*_*6.1*_), Zn (*qZn*_*6.1*_) and DTF (*qDTF*_*6.1*_) between the marker interval RM8226 and RM400 with PVE 6.6, 34.2 and 22.6% respectively. Moreover, *qFe*_*6.2*_ was found to be co-localized with *qZn*_*6.2*_ and *qDTF*_*6.2*_ between marker interval RM400 and RM162 with PVE 5.1, 14 and 10.8% respectively. Comparative analysis of data from two seasons revealed that the QTLs identified for grain iron and zinc content, namely, *qFe*_*1.1*,_ *qFe*_*1.2*,_ *qFe*_*6.1*_*, qFe*_*6.2*_, *qZn*_*1.1*_ *and qZn*_*6.2*_ (Table 4) are most significant and stable QTLs. Furthermore, comparative study was conducted to identify a similar genomic region in other mapping population and most of the reported QTLs were found to be novel except *qFe*_*1.2*_ (Table S1).

### Candidate genes associated high Fe and Zn content in grains

**Figure 3:**
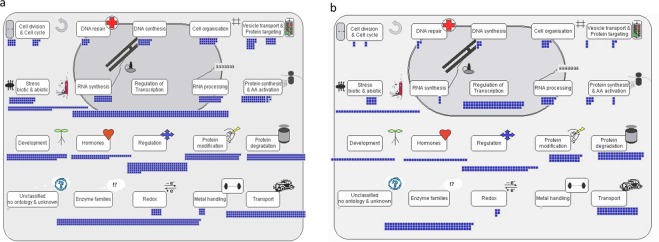
Functional annotation and categorization of genes present within (**a**) *qFe*_*1.2*_*; qZn*_*1.1*_ and (**b**) *qFe*_*6.1*_. Several genes associated with cell division, DNA repair, DNA synthesis, cell organization, vesicle transport, protein targeting, stress responsiveness, RNA synthesis, regulation of transcription, RNA processing, protein synthesis, development, hormones, regulation, protein modification & degradation, enzyme families, redox and transport were present in both the QTLs. Based on previous reports, about 34 potential candidates associated with Zn, Fe transport, homeostasis and grain content were short-listed.

The genes present within the identified QTLs region on Chr 01 and Chr 06 were retrived (Table S3), annotated and categorized broadly into cellular component, based on molecular function and with respect to the biological process (Fig. S2). On Chr 01, *qFe*_*1.1*_ possess 1495 annotated genes, while, in *qFe*_*1.2*,_ *qZn*_*1.1*_ possess 2170. In addition, the QTL detected on Chr 06 i.e., *qFe*_*6.1*_ comprised of about 815 annotated genes, (Table S2). Moreover, a comprehensive overview of functional annotation for *qFe*_*1.2*_*, qZn*_*1.1*_ and *qFe*_*6.1*_ were generated in MapMan (Fig. 3). Among several functional categories, the genes belonging to transporter activity and transcription regulator activity was first short-listed for every target QTL separately. Later, based on evidence from previous reports, about 34 potential candidates that were specific to Zn, Fe uptake, homeostasis and accumulation in grains were identified (Table S4).

### Breeding Zn and Fe bio-fortified lines with increased yield

A total of five backcross inbred lines (BILs) were identified with high Zn and moderate Fe content in grains coupled with increased yield based on the replicated data (Table S5). All the identified BILs were comparable with yield of MTU1010 (used as local yield check) for both the seasons. The grains of selected lines vary from medium slender to long slender which could be handy in breeding Zn/Fe bio-fortified lines with increased yield for diverse grain shapes (Fig. S3). Expectedly, each of the five BILs with high grain Fe and Zn content harbor at least one QTL related to grain Zn/Fe. The identified BILs could be utilized in the breeding program as a donor for developing Fe and Zn biofortified lines with better yield.

## Discussion

The lines in the mapping population displayed a wide range of variations for Fe and Zn content along with other major agronomic traits. Also, most of the traits showed normal distributions indicating a complex genetic basis. Studies have shown that complex inherited traits such as plant height, panicle length, number of tillers, days to 50% flowering, grain yield and micronutrient profile exhibit larger variation and are greatly affected by G×E^8,14^. Several earlier studies on analysis of multiple micronutrients in rice grains and mapping populations evaluated in different environments provided similar results^3,7,15,16^.

In the present report, correlation study clearly shows that there is a negative correlation between grain Fe and Zn content with yield. In addition, the strategy employed in our studies is that we select for high yielding lines with considerable level of Fe and Zn content in rice grain. There are several studies which report a positive relationship between Fe and Zn^6,17,18^, whereas strong negative correlation of grain Fe and Zn content with yield in rice ^2,7,19,20^. Therefore, it is necessary to identify high Fe/Zn donor lines with acceptable yield potential, by designing appropriate breeding strategies, selection schemes and evaluation procedures for the successful development and release of high yielding varieties with high Fe/Zn^16,21^.

Through genotype-phenotype analysis we have detected seven major QTLs for agronomic traits and four QTLs for mineral elements (Fe and Zn), majority of them were derived from the parent (Sampada). The significant QTLs identified in our study had a PV of more than 10.8%. Among identified QTLs, about 13 QTLs were consistent across the seasons, namely, *qFe*_*1.1*,_ *qFe*_*1.2*,_ *qFe*_*6.1*,_ *qFe*_*6.2*,_ *qZn*_*1.1*_*, qZn*_*6.2*,_ *qDFF*_*1.1*_*, qDFF*_*6.1*_*, qDFF*_*6.2*_*, qPH*_*1.1*,_ *qGY*_*1.1*,_ *qGY*_*11.1*_ and *qGY*_*11.2*_ were detected on Chr 01, Chr 06 and Chr 11 and these have shown effect in the background of high yielding recipient parent indicating their usefulness in the breeding program. Recent studies on QTL mapping for mineral elements in rice and other crops using RILs, ILs, F_2_, DH, MAGIC populations have identified multiple loci and clearly demonstrated the genetic complexity of the grain micronutrient traits^6,8,15^. Interestingly, out of seven reported QTLs for Fe and Zn only one QTL namely *qFe*_*1.2*_ corresponded with earlier reported QTLs^8^. Thorough characterization and validation of the reported novel QTLs/genes of the present study will be useful for performing molecular breeding.

Different mineral elements may share the same or similar pathways like protein transporters for their uptake, transport and loading. Consequently they may also share same genomic regions and QTL/genes. Several QTL clusters were identified for mineral elements and for the agronomic traits in this study. Current findings detected three co-localized QTLs responsible for different traits at specific genomic regions across Chr 01 and Chr 06. This may be due to sharing of overlapping region of QTLs or pleotropic effects. Such trait co-locations have also been reported for elements such as Cr, Mg, Cu, Si, Se, Fe, K, Mn, P^7,22,23^. Similarly, several clusters of QTLs were also found to be involved with grain minerals such as Zn, Fe, Mn, Zn/Fe, Mg, and Cu^24^. If the linked traits have positive correlations they can be simultaneously improved. However the negative linkages with grain yield must be broken before their use in breeding.

For localizing trait-related genes, QTL analysis is a robust method but identifying the causal gene(s) is a major challenge. Stable QTLs identified in the present study were used for the identification of potential candidate genes. In line with our expectation, some of the previously reported key genes associated with Zn, Fe uptake, homeostasis and accumulation in grains were present within the identified QTL regions. *OsZIP1* (LOC_Os01g74110), which is essential for Zn^2+^ influx into cytoplasm was found in our study within *qFe*_*6.1*_^25^. Interestingly, *OsZIP1* is reported to be associated with high Zn content in grain^26^. *qFe*_*6.1*_ also harbors another transporter belonging to Zip family, *OsZIP4* (LOC_Os01g66690) was proved to have higher expression in flag leaves, hence, attributing to higher Zn content in grains^26^. Besides, about five *bZIP* transcription factors were found, out of which one belongs to *qFe*_*1.1*_, a couple of them each in *qFe*_*1.2;*_ *qZn*_*1.1*_ and *qFe*_*6.4*_. Earlier, *AtbZIP19* and *AtbZIP23* transcription factors were found to be responsive under zinc-deficient condition^27^. However, their role to influence grain Zn content remains largely unknown. Also, we found vacuolar protein sorting-associated protein 28 (LOC_Os01g57260.1), vacuolar protein sorting-associated protein 53 (LOC_Os01g67880.1) in *qFe*_*1.1*_ and *qFe*_*1.2*_ *qZn*_*1.1*_ respectively. Recently, vacuolar protein sorting-associated proteins were reported to influence seed Zn content in chickpea^28^.

Moreover, *qFe*_*6.1*_ harbors ferroportin1 domain-containing protein (LOC_Os06g36450.1) which plays a vital role in vascular loading of iron in the model dicot, *Arabidopsis thaliana*^29^. Earlier reports indicate that OPTs (Oligopeptide transporters) along with YSL (Yellow Stripe like) proteins are involved in vascular translocation of Fe^2+^-NA complexes, thus facilitating iron homeostasis^25^. *OsPOT* (LOC_Os01g65110.1) was present in *qFe*_*1.1*_. Also, about 21 other POT (Proton dependent Oligopeptide transporters) genes in total were present across the identified QTLs. In the current study, about eight MATE efflux transporters were present across the identified QTLs. *OsFRD3*, a MATE transporter was previously proved to facilitate Fe transport from root to shoot^25^. Lately, AUX/IAA protein was found to impact seed iron content in chickpea^25^. In the current study, two auxin-responsive transcription factors namely, *OsIAA5* and *OsIAA6* were found within the identified QTLs.

Meta-analysis provided insights into the overall consensus genomic regions based on the previous reports for grain Fe ans Zn content. The analysis performed using Biomercator V4.2^30^ resulted in three meta-QTLs for grain Fe and two meta-QTLs for grain zinc (Fig. S4). Meta-QTLs were observed on only two chromosomes namely Chr 07 and Chr 12 (Table S6). On Chr 07 meta-QTL, *MFeQTL7.1* with confidence interval of 16.3 cM (corresponded to two QTL with PVE up to 69%) and for zinc *MZnQTL7.1* with confidence interval of 30.63 cM (corresponded to three QTL with PVE ranging from 11.4 to 35%) was observed. Similarly, three meta-QTLs were obtained on Chr 12, two for grain Fe, *MFeQTL12.1* (corresponded to two QTL with PVE ranging from 16.9 to 71%) with confidence interval of 28.16 cM and *MFeQTL12.2* (corresponded to two QTL with PVE ranging from 13.8 to 71%) with confidence interval of 8.55 cM. For grain Zn, *MZnQTL12.1* with confidence interval of 18.22 cM (corresponded to two QTL with PVE ranging from 34 to 35%) was obtained. Further, the identified meta-QTLs holds promise for the development of biofortified versions of rice. As the QTLs reported in our study are novel, no meta-QTLs were found in that region. The novel QTLs identified in our study along with these meta-QTL regions are expected to aid in developing biofortified rice lines.

The identified potential candidate genomic regions/candidate genes are expected to play a vital role for achieving high Fe and Zn content in rice grains. Lines with higher Fe and Zn will be tested with inductively coupled plasma mass spectroscopy (ICP-MS) method for further confirmation of identified lines for future study. Further characterization of these genomic regions/candidate genes would uncover the molecular basis of grain Fe and Zn accumulation. In addition, the present study identified several breeding lines with high grain Zn and grain yield across the seasons. About five lines were identified with Fe more than 13 ppm; Zn more than 21 ppm with better yield and good agronomic traits in this study. Further these lines can be used as donors in the future breeding programs or can be directly tested in multi-location trials for commercial cultivation after proper testing.

## Conclusion

In conclusion, a total of 20 QTLs were detected for agronomic and nutritional traits through composite interval mapping. Comparative analysis revealed two major QTLs (upto 17.1% PVE) for Fe and three major QTLs (upto 34.2% PVE) for Zn. Analyzing QTLs for mineral content and yield components together revealed 12 co-localized QTLs responsible for different traits at specific genomic regions across Chr 01, Chr 06 and Chr 11. Co-localized QTLs on Chr 01 and Chr 06 possessed candidate genes such *as OsPOT, OsZIP4, OsFDR3, OsIAA5* etc., which are likely to influence grain Fe and Zn accumulation. Additionally, meta-analysis of grain iron and zinc QTLs helped to identify precise meta-QTLs. Most of the identifed QTLs were novel and can be deployed in MAS for development of superior versions of rice lines with high yield coupled with high grain Fe and Zn content.

## Materials and Methods

### Development of mapping population

The experimental material consists of 111 lines derived from BC_2_F_5_ mapping population of RP Bio-226/Sampada developed at Indian Institute of Rice Research (IIRR), Hyderabad. Improved Samba Mahsuri (RP-Bio226) is a high yielding fine grain rice variety with 135–140 days duration developed using marker-assisted selection and has three major bacterial blight resistance genes *Xa21, xa13* and *xa5*. Sampada (DRR Dhan37) is a medium duration variety maturing in about 130–135 days. Both of these varieties are high yielding and are released for commercial cultivation by Indian Institute of Rice Research (IIRR), Hyderabad, India.

### Phenotyping of mapping population

Experiment was conducted in alpha lattice design and the phenotypic data was generated from DS2015 and WS2015 of 111 lines along with two parents (RP-Bio226 and Sampada) and check (MTU1010), in two replications. Mapping population was evaluated in DS2015 and WS2015 in alpha lattice design with two replications. Several key traits such as, days to 50% flowering (DFF), plant height (cm), panicle length (PL), number of tillers (NT), yield/plot (kg/ha) were recorded. Micronutrient analysis from grains was performed using XRF (S2 RANGER, Germany) at Harvest Plus, ICRISAT, Hyderabad facility. XRF works on the principle of non-destructive emission, in which the atoms are excited by an external energy source, emit X-ray photons of a characteristic wave length, and by counting the number of photons of each energy emitted from a sample, the elements present may be quantified (ppm). For iron and zinc analysis, rice grain was dehusked using de-husker (H-750, Krishi International, India). The dehusked rice grains carry a mixture of whole and broken grains, whole grains were separated from the broken ones, and then the brown rice was analyzed for the minerals (Fe and Zn) using XRF spectrometry. Previous reports suggest that in polished rice the zinc and iron content generally reduce by 3 and 7 times respectively because of removal of aleurone layer and embryo^31^. The experiments for phenotyping were conducted in the field with alfisol soil type (Table S7).

### DNA isolation and parental polymorphism

About 200 mg of leave sample collected from individual plants in the mapping population (111 individual), and parental lines were used to isolate genomic DNA using IRRI protocol (TPS buffer) and was finally diluted in 20 µl TE buffer. The quality of the isolated DNA was confirmed on 0.8% agarose gel. About, 10 ng DNA from each sample was used for PCR. Parental polymorphism was surveyed using 1000 SSR markers which was resolved in 4% agarose gel. The identified polymorphic SSR markers between parents with good difference (>10 bp) was used to screen the mapping population. Genotypic data generated after scoring of mapping population was used for the construction of linkage map.

### Construction of genetic maps

The genetic maps were constructed using QTL IciMapping version 4.1. The grouping and ordering of markers were carried out using regression mapping algorithm with a maximum recombination frequency of 0.4 at minimum logarithm of odds (LOD) value of 2.5 using the command “LOD groupings” and “create groups for mapping” into respective linkage groups (LG). Kosambi map function was used for the construction of genetic map and calculation of map distance from recombination fractions. After developing the framework genetic maps with the marker orders, the unmapped markers were integrated into different linkage groups at recombination frequency up to 50% using ripple command. The resultant genetic maps were visualized using IciMapping version 4.1.

### QTL analysis and visualization

QTL analysis was conducted with QTL cartographer version 2.5 using CIM. The location of each QTL was determined according to its LOD peak location and the surrounding region. The LOD score values (2.5) were used to determine the significance of QTL. A QTL is named as *qFe1.2* with ‘Fe’ being the trait abbreviation grain iron, ‘1’ is the chromosome number, ‘2’ the number to specify different QTLs for the same trait in one linkage group based on the physical positions of left and right QTL linked markers. MapChart 2.30 was used to project QTLs on the linkage groups^32^.

### Identification and analysis of candidate genes within the QTLs

The candidate genes within the identified QTL region was retrieved using IRRI galaxy resource (http://galaxy.irri.org/). Following it, they were functionally characterized into various categories using WEGO^33^ and were visualized in MapMan 3.6.0 RC1^34^. Further, literature mining was performed to short-list the potential candidate genes related to Zn, Fe uptake, homeostasis and accumulation in grains, that are present within the identified QTLs.

## Supplementary information

Table S3
